# Dietary supplementation with pterostilbene activates the PI3K-AKT-mTOR signalling pathway to alleviate progressive oxidative stress and promote placental nutrient transport

**DOI:** 10.1186/s40104-024-01090-9

**Published:** 2024-10-06

**Authors:** Mingming Cao, Liyun Bai, Haoyun Wei, Yantong Guo, Guodong Sun, Haoyang Sun, Baoming Shi

**Affiliations:** College of Animal Science and Technology, Northeast Agricultural University, Harbin, 150030 PR China

**Keywords:** Nutrient transporters, Placenta, Progressive oxidative stress, Pterostilbene, Sows

## Abstract

**Background:**

Progressive oxidative stress easily occurs as a result of a gradual increase in the intensity of maternal metabolism due to rapid foetal development and increased intensity of lactation. However, studies on the effects of processive oxidative stress on nutrient transport in the placenta have received little attention. The present study was conducted on sows at 85 days of gestation to study the effects of pterostilbene (PTE) on maternal oxidative stress status and placental nutrient transport.

**Results:**

PTE increased the antioxidant capacity and immunoglobulin content in mothers’ blood and milk, reduced the level of inflammatory factors, and improved the nutrient content of milk. PTE also reduced sow backfat loss and the number of weak sons, and increased piglet weaning weight and total weaning litter weight. We subsequently found that PTE enhanced placental glucose and fatty acid transport and further affected glycolipid metabolism by increasing the expression of *LAL*, *PYGM*, and *Gbe-1*, which activated the *PI3K* phosphorylation pathway. Moreover, PTE addition altered the relative abundance of the Firmicutes, Proteobacteria, *Parabacillus*, and *Bacteroidetes-like RF16 groups* in sow faeces. PTE increased the levels of acetate, propionate, butyrate and isovalerate in the faeces.

**Conclusions:**

These findings reveal that the addition of PTE during pregnancy and lactation mitigates the effects of processive oxidative stress on offspring development by altering maternal microbial and placental nutrient transport capacity.

**Graphical Abstract:**

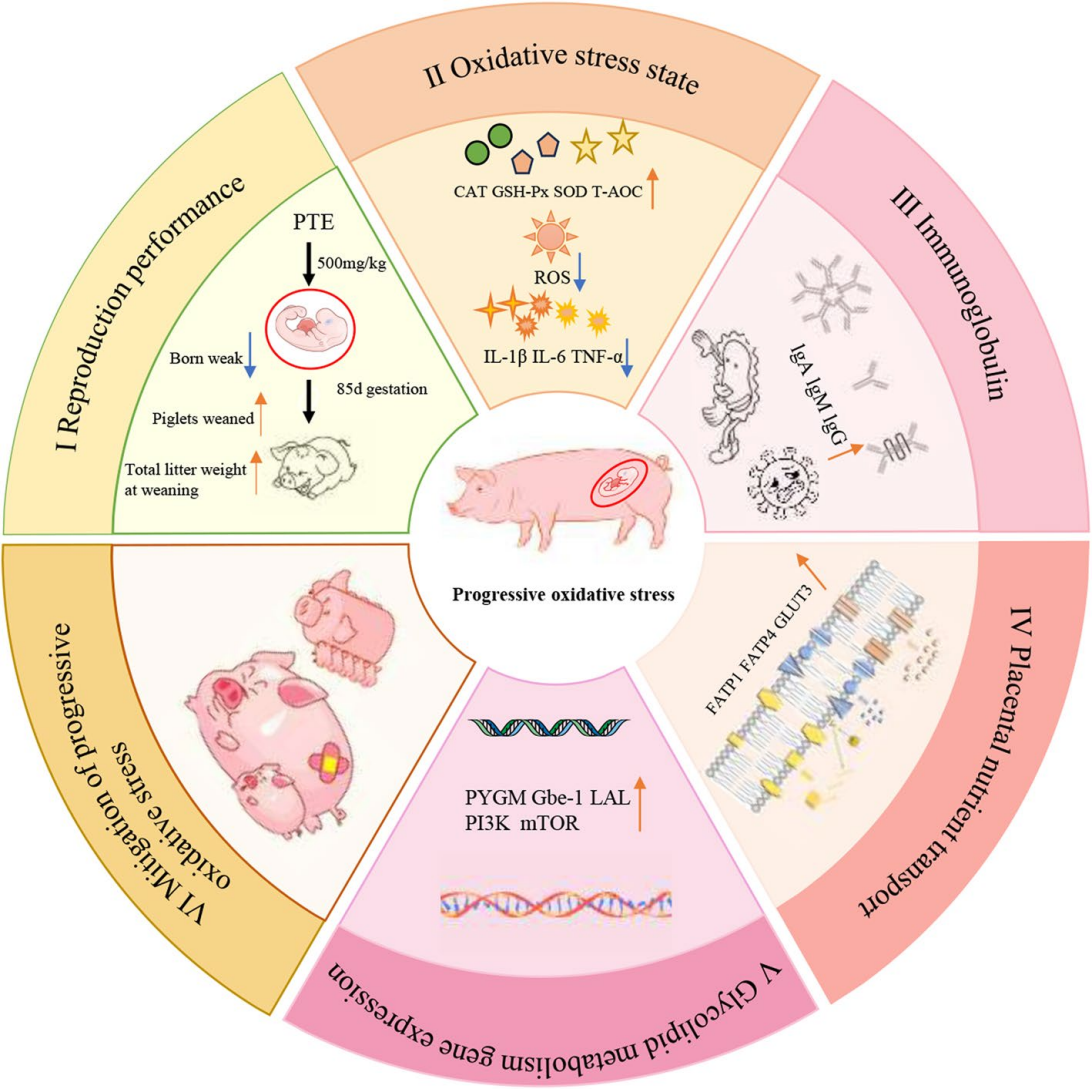

**Supplementary Information:**

The online version contains supplementary material available at 10.1186/s40104-024-01090-9.

## Introduction

Normal pregnancy is a complex, irreversible, but highly programmed series of processual events, including implantation, decidualization, placental development, and partum [1, 2]. The smooth development of the chronological sequence is crucial for a normal pregnancy, and any unfavourable factors may affect the health of the mother and the normal development of the foetus [3]. The development of foetal tissues and organs requires an adequate supply of nutrients and oxygen. However, the slower rate of foetal development and lower nutritional needs during early pregnancy are supported by tissue nutrition, primarily by the endometrial glands, to support embryonic and early placental development [4]. The foetus enters a stage of rapid development in late pregnancy, and to adapt to this physiological change in the foetus, the establishment of the chorioallantoic placenta involves transformation of the maternal‒foetal dialogue into a haemotrophic nutrition [2]. It also facilitates the mother’s mobilization of more fat and sugar breakdown, and the release of free fatty acids, oxygen, glucose, and other nutrients into the body’s circulation to be passed on to the foetus [5]. These metabolic processes also inevitably produce lipid peroxides, O_2_^−^, H_2_O_2_ and OH^−^ as oxygen species (ROS). Progressive oxidative stress refers to the phenomenon in which female animals processively produce large amounts of ROS during the reproductive cycle, especially during the later stages of gestation, when the foetus is in the rapid developmental stage, parturition, and lactation. The placenta, the main organ for ROS production during pregnancy and the central organ for regulating ROS homeostasis, bears the brunt of this challenge [6].

The placenta is a temporary, but highly specialized organ that serves as a bridge between the mother and the foetus, not only providing nutrients, transporting gases, and excreting wastes, but also secreting hormones to provide the foetus with immune substances [7]. In addition, the nutritional needs of the foetus at the gestation stage are completely dependent on placental transporter transport, and the increase in the nutritional needs of the foetus leads to a corresponding increase in the metabolic strength of the mother to produce a large number of ROS. ROS accumulation induces damage to the placenta and macromolecular lipids and proteins, interfering with the function of transport carriers and ultimately leading to pregnancy complications and affecting the normal development of the foetus [8]. Placental nutrient transport carriers are mainly glucose transport carriers, amino acid transport carriers, and fatty acid transport carriers. Glucose transport relies mainly on the *GLUT* family [9]. Interestingly, *GLUT1* is involved mainly in transplacental transfer, whereas *GLUT3* is expressed in epithelial and mesenchymal cells and is not involved in transplacental transfer [10]. Amino acid transport relies primarily on the amino acid transporter system, including cationic, anionic and neutral amino acid transport systems [11]. Fatty acid transport may be driven by a *cis*-concentration gradient (maternal‒foetal), with uptake by the FATP family [12]. FATP1, FATP4, and CD36 are mainly responsible for the transport of long-chain polyunsaturated fatty acids [13]. Nutrient transport carriers are essential to ensure adequate nutrition for the normal development of the foetus. Protein carbonyl to nitro conversion due to progressive oxidative stress has been reported to alter protein function [14]. This alteration affects the normal performance of a variety of functions such as transporter transfer efficiency, signalling molecules, and structural proteins [15]. Previous studies have shown that prenatal stress disrupts placental function and nutrient transport systems, affecting the inadequate supply of nutrients needed for the normal formation and function of brain structures in offspring [16]. However, an in vitro study of tert butyl hydrogen peroxide induced oxidative stress in pTr cells, revealed that oxidative stress can affect the activity of the L-alanine placental transporter by inhibiting system A [17]. However, the mechanisms by which processive oxidative stress affects placental nutrient transport capacity and regulates foetal development have not been reported.

Pterostilbene (PTE) is a diphenylethylene compound found in many natural products and is also known as 3,5-dimethoxy-4′-hydroxystyrene [18]. It is widespread in blueberries (*Vaccinium*), Dragon's blood and grapes (*Vitis *spp.) [19]. Many animal and cellular experiments have demonstrated the anti-inflammatory, hypoglycaemic and antioxidant activities of PTE [19, 20]. A recent cell experiment, confirmed that PTE can alleviate H_2_O_2_ induced oxidative damage by activating the Nrf2 pathway in ovarian granulosa cells [21]. In addition, Malik et al. [22] confirmed that PTE can exert antioxidant effects by reducing lipid accumulation in HepG2 cells and alleviating lipid oxidative damage. In a study of gestational diabetic mice, PTE was found to normalize the circulating concentrations of VLDL and LDL in the blood and alleviate lipid peroxidation caused by disorders of lipid metabolism [23]. PTE can mitigate oxidative damage caused by processive oxidative stress in mothers. PTE is a dimethyl analogue of resveratrol. Resveratrol is a natural compound extracted from wine with similar antioxidant and anti-inflammatory activities [24]. Because the 2 hydrogen ions on it are replaced by methyl groups, PTE exhibited superior bioavailability and lipid solubility [25]. In animal studies, PTE was found to be four times more bioavailable than resveratrol [26]. These findings suggest that PTE has potential as a promising and effective dietary supplement.

Studies have shown that the placenta at 85 days of gestation also has a strong angiogenic response and that the foetus enters a rapid developmental stage. Therefore, we studied sows at 85 days of gestation [27]. The oxidative stress status during the gestational phase was evaluated, and the mitigating effect of PTE was assessed by testing the antioxidant capacity of maternal blood and milk. In addition, we analysed plasma biochemical indices, milk nutrients, plasma and milk inflammatory factors, immune factors, placental nutrient transporter function, sow reproductive performance and piglet health.

## Materials and methods

The feeding and treatment of sows in this study were performed in strict accordance with the regulations on the management of experimental animals in Heilongjiang Province, China (revised in 2016). All animal experiments were approved by the Animal Welfare Committee of Northeast Agricultural University (# NEAU‒2013).

### Chemicals

Pterostilbene (S26817‒100 g; molecular weight: 256.3; purity: 97%) was obtained from Shanghai Yuanye Biological Co., Ltd. (Shanghai, China). The chemical structure of pterostilbene (C_16_H_16_O_3_) is shown in Fig. S1. Pterostilbene purpureus concentrations were chosen on the basis of previous studies [28].

### Animals and experimental design

Twenty healthy, close-weight Landrace × Large White crossbred pregnant sows with a parity of 2 to 4 litters were selected. All sows were randomized into 2 treatment groups of 10 sows each. The control group was fed a basic diet, and the PTE group was fed a basic diet supplemented with 500 mg/kg PTE. The experiment starts at 85 days of pregnancy and ends at 21 days of weaning for piglets. During pregnancy, sows live within individual limits (0.7 m × 2.1 m) and are transferred to the parturition room 7 d before the expected date of parturition. The birthing room is equipped with electric heating plates and heating lamps to ensure that the piglets live in a suitable environment, and the piglets are weaned at 21 days of age. Pregnancy and lactation feeds are strictly formulated according to NRC (2012) standards, and the composition and nutrient levels are shown in Table S1 [29]. The number of litters, live births, born weak, stillbirths, dry carcasses and birth weight, as well as the number of weaned piglets and weaned piglet weight, were recorded to facilitate the analysis of the breeding performance of the sows. Piglets with a birth weight less than the average litter live weight were considered born weak. Body weight and backfat thickness were recorded at 107 d gestation, 7 d postpartum, and at weaning in the sows. Sow feed intake was recorded in 2 stages, from gestation d 85 to 107 and from gestation d 107 to weaning, and the average daily feed intake was calculated.

### Sample collection and preparation

Blood samples were collected via the marginal ear vein method from six randomly selected sows in each treatment group 7 d prior to expected farrowing, on the day of parturition, on the d 21 of lactation and prior to morning feeding. The collected blood samples were placed in a centrifuge at 3,000 × *g* for 15 min, and the separated plasma was placed in a −20 °C refrigerator for storage. At the time of parturition, a placenta was randomly selected and 10–20 g of sample was immediately collected from 3 cm from the umbilical cord (avoiding dense blood vessels; near the piglet side). Placental tissue (0.8 cm^2^) was fixed in 4% paraformaldehyde solution for HE staining and IF, and the remaining tissues were collected into tubes and stored in liquid nitrogen. Approximately 15–25 mL of breastmilk was collected from different parts of the sow’s front, middle, and hind teats on the day of parturition and in the morning before being fed on the day of weaning. Sow foeces were collected on the 18^th^ day of lactation for three consecutive days and stored in a refrigerator at −20 °C.

### Measurement of plasma biochemical indicators

Sow plasma samples were analysed via a fully automated Beckman Coulter biochemical analyser (Unicel D×C 800 Synchron Clinical System). BUN, UA, GLU, ALT, AST, ALP, TBA, TBI, TP, ALB, GLOB, A/G, LD, TG, TC, CHOL, HDL-C, and LDL-C were purchased from Nanjing Jiancheng Bioengineering Institute (Nanjing, China), and all the analyses were carried out in strict accordance with the instructions.

### Oxidative stress assessment

The levels of the antioxidant indicators T-AOC, MDA, SOD, GSH-PX, and CAT in plasma and breast milk were measured via commercially available kits purchased from Nanjing Jiancheng Bioengineering Institute (Nanjing, China), according to the manufacturers’ instruction. All measurements were normalized for processing.

### Inflammatory mediators and immunoglobulin assay

The levels of the proinflammatory factors IL-1β, IL-6, and TNF-α; the inflammatory factor IL-10; and the immunoglobulins IgA, IgG, and IgM in the plasma and breast milk were determined via ELISA. Commercial reagent kits were purchased from Shanghai Yuanju Biotechnology Center (Shanghai, China). The test procedure was carried out in strict accordance with the instructions provided by the reagent vendor. In brief, the samples were added to a microtiter plate and incubated at 37 °C for 60 min to allow the samples to fully react with the enzyme. The mixture was washed with washing buffer 5 times, in the dark at 37 °C for 15 min, the reaction termination solution was added, and the absorbance was detected at 405 nm.

### Immunofluorescence examination

Sections of the produced placental wax blocks were used for IF staining for fatty acid transporter protein 1 (FATP1) and glucose transporter protein 3 (GLUT3). Anti-FATP1 antibody (Abclonal WH342075) was purchased from Abclonal (Wuhan, China). Anti-GLUT3 (Wanlei WL05064) antibody was purchased from Wanleibio (Shenyang, China). Three randomly selected fields of view for each produced slide were observed with a fluorescence microscope (EVOS M700, Jena, Germany) and quantitatively analyzed.

###  Quantitative real-time PCR analysis

A total of 0.1 g of placental tissue was accurately weighed, and 1 mL of TRIzol reagent was added to each sample to extract total RNA. The samples were solubilized via DEPC water after cryogenic milling, lysis, layering, clustering, and washing. The total RNA concentration and purity were determined via an ultramicro spectrophotometer (Implen GmbH, Munich, Germany). The minimum concentration of total RNA was greater than 500 ng/µL, and the A_260_/A_280_ ratio was between 1.8 and 2.0. The eligible RNA was reverse transcribed to cDNA via 5× Integrated RT MasterMix according to the manufacturer’s instructions (DiNing, Beijing, China). Then, qRT-PCR was performed according to the instructions (Takara, Dalian, China). The qRT-PCR primers used were designed and synthesized as shown in Table S2. The primers used were designed on the basis of the CDS of the SUS scrofa gene found on NCBI [30]. The results were used to calculate the relative expression of mRNAs via the 2^−ΔΔCt^ method.

### Western blotting

A total of 0.2 g of placental tissue was accurately weighed, and total placental protein was extracted with RIPA lysis buffer (Beyotime, Shanghai, China). The lysed samples were centrifuged at 12,000 r/min at 4 °C for 15 min, and the supernatant was aspirated. A portion of the sample was removed to determine the protein content of the sample, and the BCA protein assay kit (Epizyme Biotech, Shanghai, China) was used to calculate the injection volume of the sample accurately. The remaining samples were added to 4 volumes of SDS‒PAGE buffer (Epizyme Biotech, Shanghai, China) and heating at 90 °C for 15 min. Proteins from the samples were separated via SDS‒PAGE, and matched gel slices were placed onto polyvinylidene difluoride (PVDF) membranes. The proteins were transferred to an antibody incubation cassette and washed once with TBST. Then, the samples were blocked with 5× protein-free blocking solution (Epizyme Biotech, Shanghai, China) for 10 min, and the diluted primary antibody was added to the antibody incubation cassette and incubated for 12 h at 4 °C in the refrigerator. The PVDF membrane was washed 5 times with 1× TBST, incubated with the diluted secondary antibody at 37 °C for 1 h, and then washed 5 times with 1× TBST. Antibody-tagged proteins were visualized via a gel imaging and analysis system (Alpha Innotech Corporation, CA, USA) and quantified via ImageJ (NIH, USA). Table S3 contains information on the antibodies used in this study.

### Determination of short-chain fatty acids

Two grams of sow faeces were accurately weighed, and the samples were soaked in 0.5% metaphosphoric acid solution and mixed thoroughly. The resulting sample was centrifuged at 4 °C for 20 min at 12,000 r/min, and the supernatant was collected in a new tube. The supernatant was filtered twice through a 0.22-μm filter to remove particulate impurities in the supernatant. The prepared samples were placed into a gas chromatography machine for testing. Prior to sample testing, a standard curve with SCFAs standards was generated to calculate the concentration of each SCFA in the prepared samples. The ratio of the peak area of the standard and the internal standard was used as the vertical coordinate, and the ratio of the number of the standard and the internal standard was used as the horizontal coordinate to plot the standard curve.

### 16S rDNA gene sequencing bioinformatics analysis

The samples were sequenced via the NovaSeq PE250 platform, where the samples were assigned to pairs on the basis of their unique identifiers and assembled via FLASH end-reads. The raw reads under the filtering conditions were filtered according to fqtrim (version 0.94) to obtain clean data. Denoising, splicing, and filtering chimaeras on clean data via DADA2. The samples were then species-annotated via QIIME2 software in conjunction with the SILVA database. The alpha and beta diversity indices were tallied via QIIME2. The sequences were compared via BLAST, and the featured sequences were annotated via SILVA. Differential expression of bacterial colonies between 2 groups of samples assessed by LEfSe.

### Statistical analysis

All the data were analysed via the two-tailed unpaired *t*-test in IBM SPSS (Version 27.0, IBM Corp., Armonk, NY, USA). Statistics, calculations and categorization of raw data were performed via Microsoft Excel. All the data obtained are expressed as the mean ± SD. ^*^P < 0.05 indicates significant differences, and ^**^P < 0.01 indicates highly significant differences. In addition, the correlation between faecal flora and factors related to nutrient digestion and transportation was investigated via Spearman’s analysis. GraphPad Prism (version 9.5.1, USA) was used to plot the results.

## Results

### Effect of PTE on the reproductive performance of sows

**Fig. 1:**
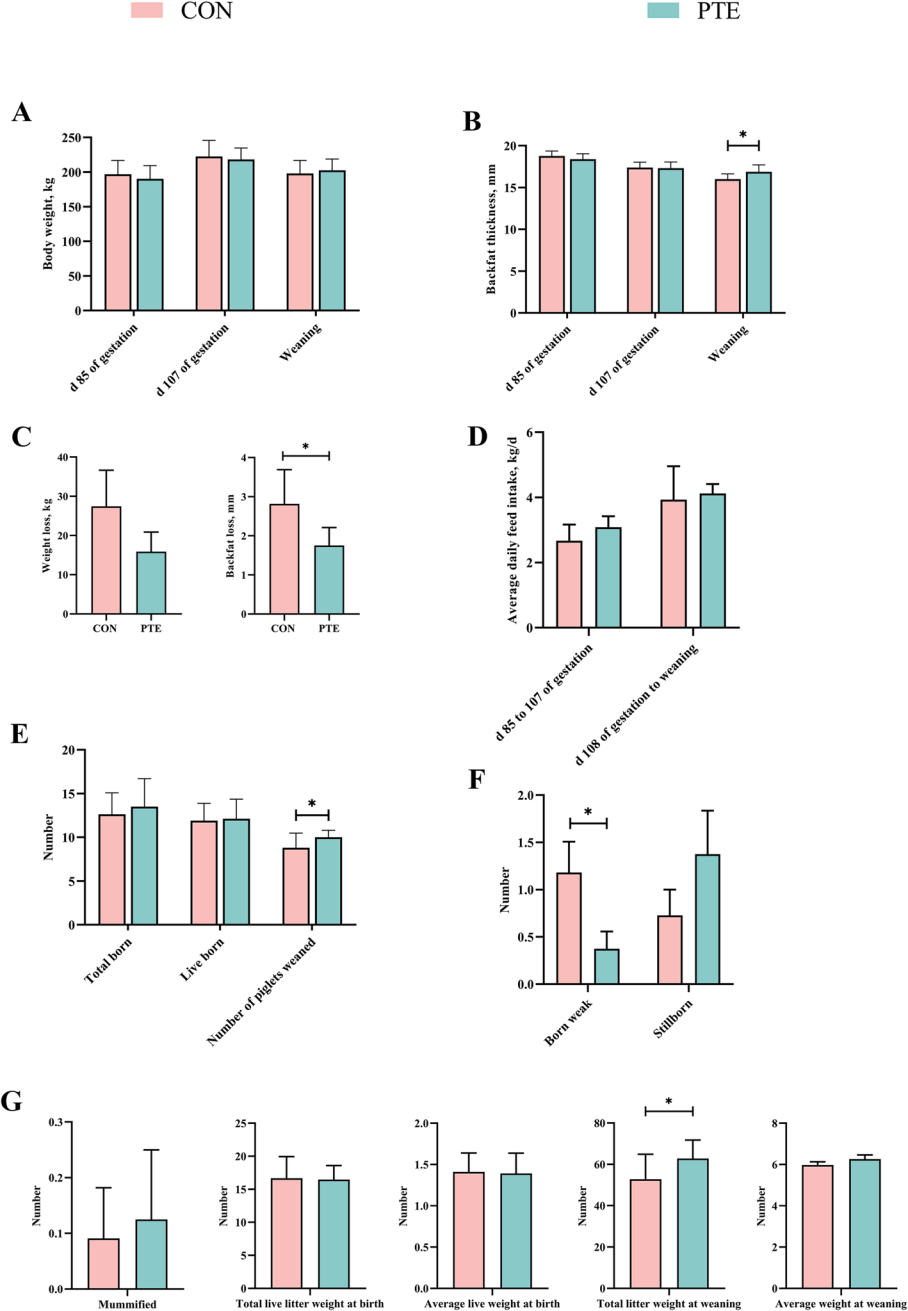
Effect of PTE on reproductive performance of sows (**A**–**G**). CON: control group; PTE: Pterostilbene group. Data are expressed as mean ± SD (*n* = 10 for each group). ^*^P < 0.05, ^**^P < 0.01 compared to the control group

As shown in Fig. 1A–G, differences in sow body weight and backfat thickness at d 85 and 107 of pregnancy and at weaning were not significant (*P* > 0.05). Importantly, backfat loss in sows during this phase was significantly lower (*P* < 0.05) due to the addition of PTE. The average daily feed intake from d 85 to 107 of pregnancy and from d 107 of pregnancy to weaning was also not significantly different (*P* > 0.05). The total birth weight, number of live births, number of stillborn, number of dry carcasses, individual birth weight, and average weaning weight were not significantly different (*P* > 0.05). Notably, the addition of PTE significantly decreased the number of born weak piglets and increased the number of weaned piglets and total weaned litter weight (*P* < 0.05).

### Effects of PTE on plasma biochemistry, antioxidant indices, and immunoglobulin and inflammatory factor levels in sows

**Fig. 2:**
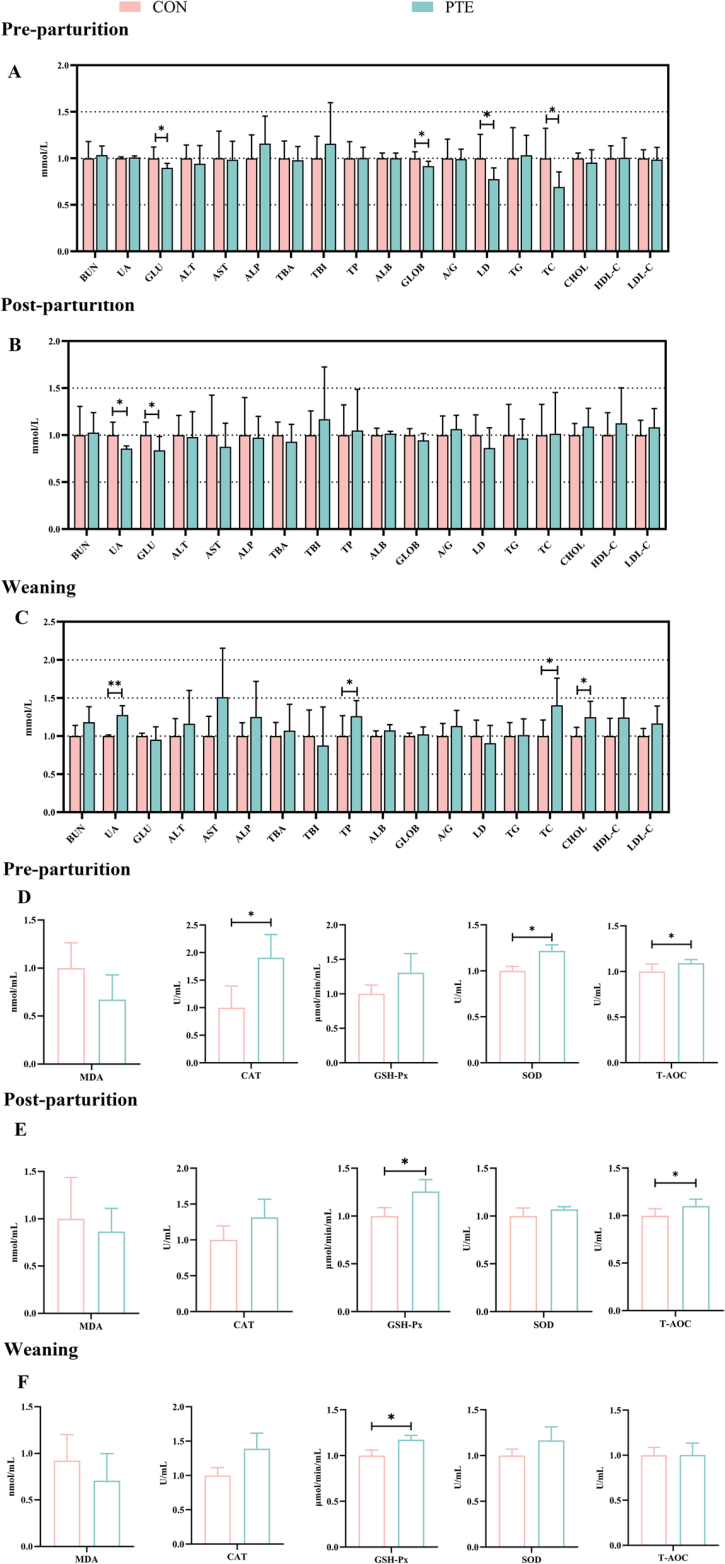
Effect of PTE on blood biochemistry, antioxidant ability in sows. **A**–**C** Serum biochemicals 7 d before parturition, on the day of parturition, and on the day of weaning. **D**–**F** Serum antioxidant indices 7 d before parturition, on the day of parturition and on the day of weaning. CON: control group; PTE: Pterostilbene group. Data are expressed as mean ± SD (*n* = 6 for each group). ^*^P < 0.05, compared to the control group

**Fig. 3:**
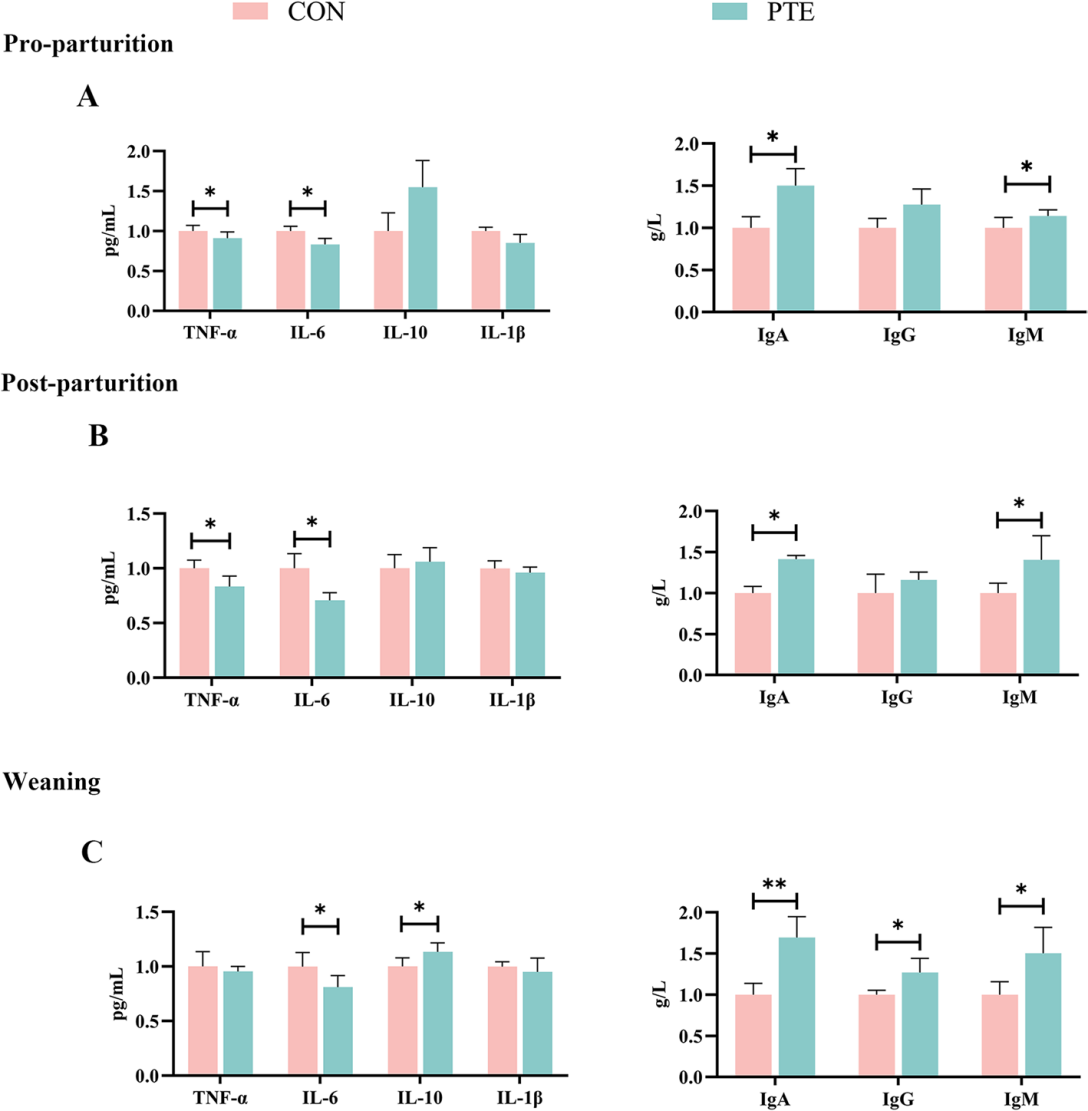
Effect of PTE on blood levels of inflammatory factors and immunoglobulins. **A**–**C** Serum inflammatory factor and immunoglobulin levels 7 d before parturition, on the day of parturition, and on the day of weaning. CON: control group; PTE: Pterostilbene group. Data are expressed as mean ± SD (*n* = 6 for each group). ^*^P < 0.05, ^**^P < 0.01 compared to the control group

As shown in Fig. 2A, D and 3A, the differences in the plasma levels of GLU, GLOB, LD, TC, CAT, SOD, TNF-α, IL-6, IgA, and IgM in the PTE group on the d 7 of the prenatal period were statistically significant (*P* < 0.05). Unfortunately, there was no statistically significant effect on other relevant indicators (*P* > 0.05). Moreover, the plasma levels of UA, GLU, TNF-α, and IL-6 were significantly lower (*P* < 0.05), and the levels of GSH-Px, T-AOC, IgA, and IgM were significantly greater (*P* < 0.05) on the day of delivery in the PTE group (*P* < 0.05; Fig. 2B, E and 3B). In the plasma PTE group on the day of weaning, the levels of UA, TP, TC, CHOL, GSH-Px, IL-10, IgA, IgG, and IgM were greater (*P* < 0.05), and the levels of IL-6 were lower (*P* < 0.05; Fig. 2C, F and 3C).

### Effect of PTE on the nutrient transport function of the sow placenta

**Fig. 4:**
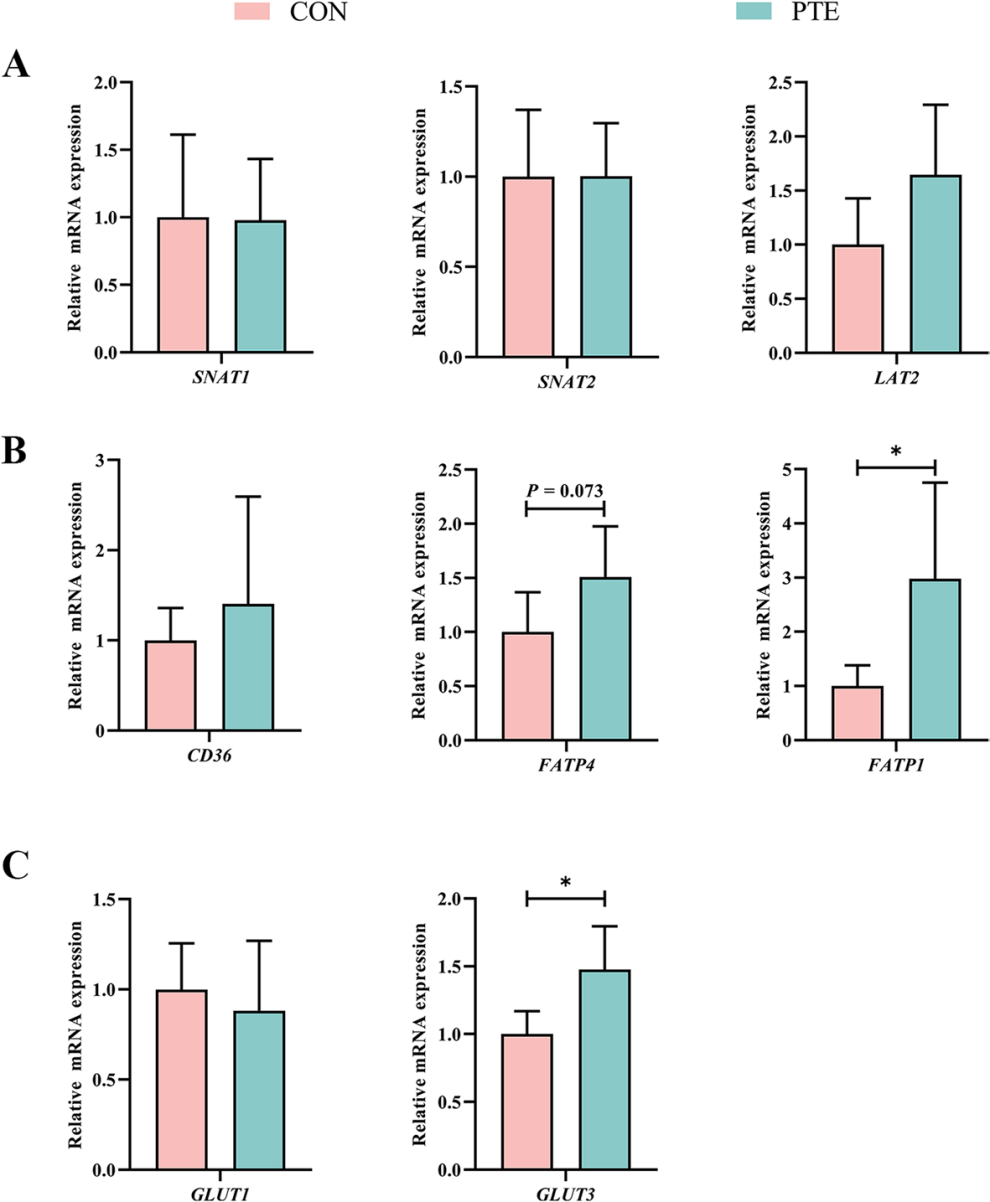
Effect of PTE on placental nutrient transport capacity. **A** Amino acid transporter mRNA expression. **B** Expression of lipid transporter mRNAs. **C** Glucose transporter mRNA expression. CON: control group; PTE: Pterostilbene group. Data are expressed as mean ± SD (*n* = 6 for each group). ^*^P < 0.05, compared to the control group

**Fig. 5:**
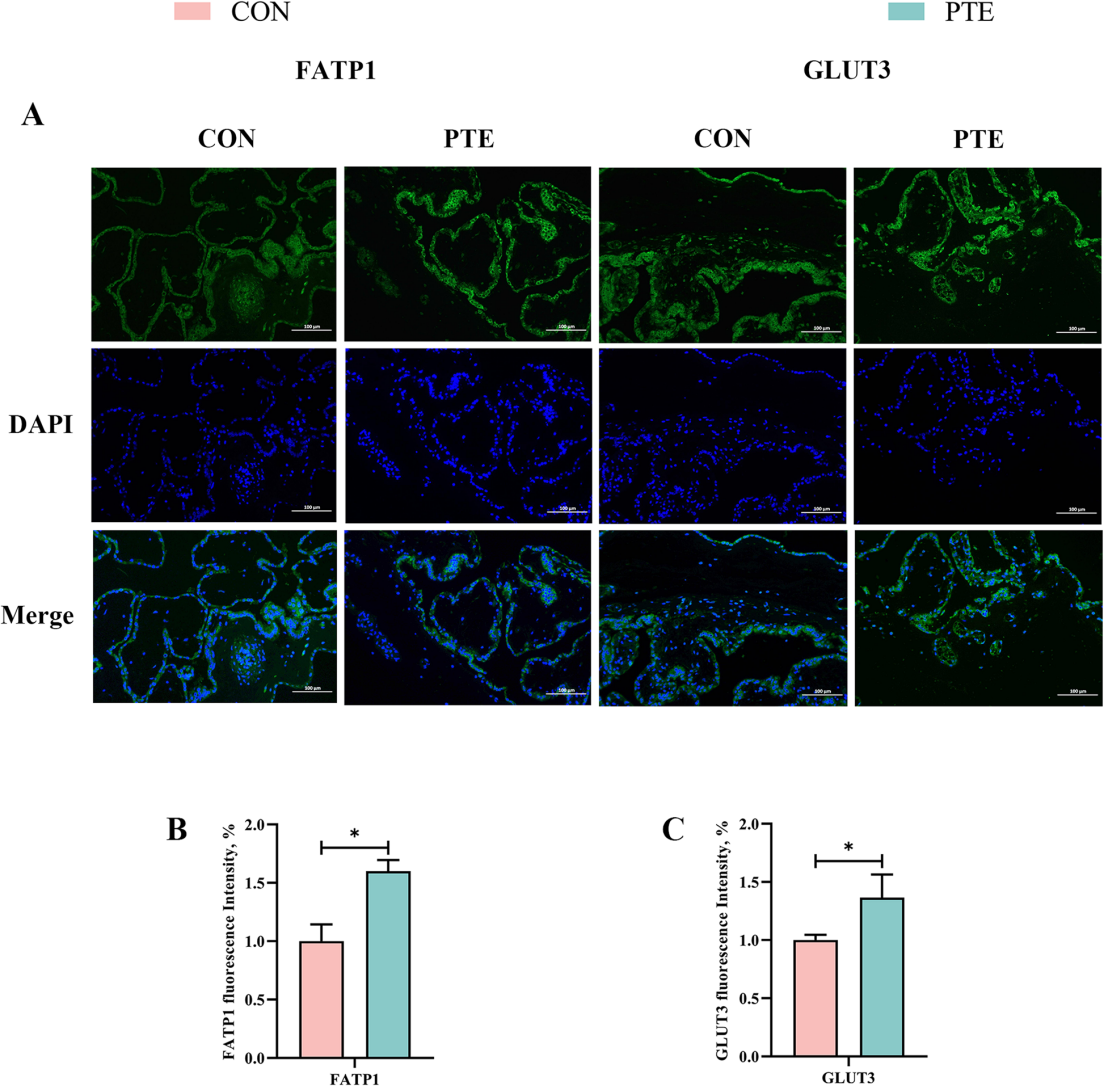
Effect of PTE on placental nutrient transport capacity. **A**–**C** Fatty acid transporter FATP1 and glucose transporter GLUT3, 200× analysis of IF staining. Data are expressed as mean ± SD (*n* = 3 for each group). ^*^P < 0.05, compared to the control group

Oxidative stress affects maternal nutrient transport. We therefore investigated the expression of mRNAs related to nutrient transport in the maternal placenta to explore the effects of processive oxidative stress on nutrient transport function. As shown in Fig. 4A–C, PTE had no significant effect on *SNAT1*, *SNAT2*, *LAT2*,* CD36*, or *GLUT1* (*P* > 0.05) but tended to increase the expression of *FATP4* (*P* = 0.073). In addition, IF staining (Fig. 5A–C) revealed that the addition of PTE increased the expression of fatty acids and glucose transporters (*P* < 0.05), which was also confirmed by qRT‒PCR. These findings suggest that maternal oxidative stress interferes with nutrient transport and the addition of PTE protects the transporter from these effects.

### Effect of PTE on placental energy metabolism in sows

**Fig. 6:**
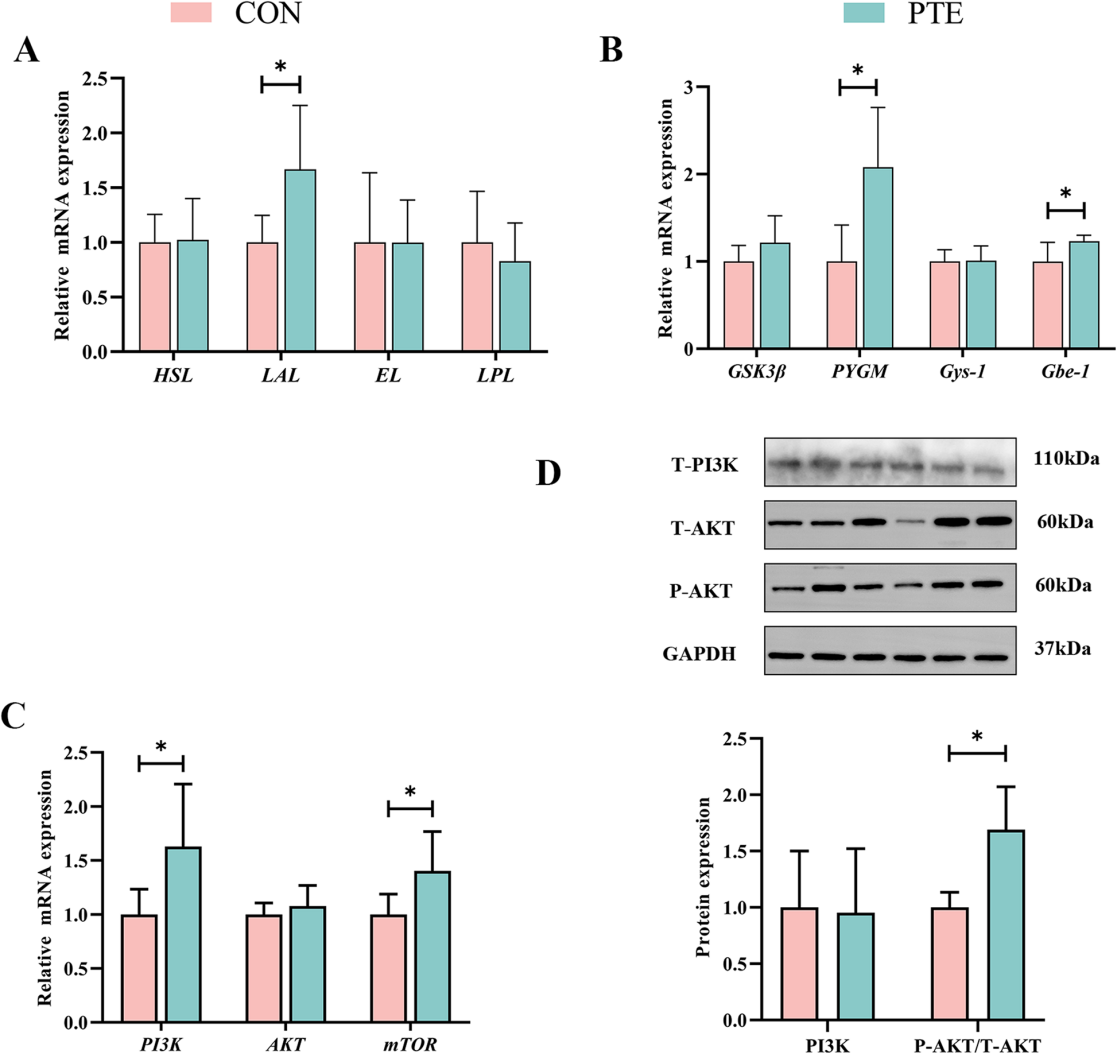
Effect of PTE on placental glycolipid metabolism. **A** Lipid metabolism catalase mRNA expression. **B** Glucose metabolism catalase mRNA expression. **C** and **D** PI3K pathway mRNA and protein expression. CON: control group; PTE: Pterostilbene group. Data are expressed as mean ± SD (*n* = 6 for each group). ^*^P < 0.05, compared to the control group

The results are shown in Fig. 6A and B. The relative expression levels of *PYGM*, *Gbe-1* and *LAL* were significantly increased in the PTE group (*P* < 0.05), but there were no statistically significant differences in any other related indices (*P* > 0.05). Additionally, we also examined the expression levels of signalling pathways that regulate glycolipid metabolism. The PTE group presented significantly increased expression of *PI3K* and *mTOR* (*P* < 0.05), but no significant change in *AKT* (*P* > 0.05). This finding was also confirmed by the results of the Western blot analysis, which revealed that T-PI3K protein expression was not significantly changed by the addition of PTE (*P* > 0.05), but the T-AKT/P-AKT ratio was increased (*P* < 0.05; Fig. 6C and D). These findings indicate that PTE addition activates the PI3K-AKT-mTOR phosphorylation pathway, which participates in the regulation of placental nutrient transport and metabolism.

### Effects of PTE on breast milk composition, antioxidant indices, and immunoglobulin and inflammatory mediator levels in sows

**Fig. 7:**
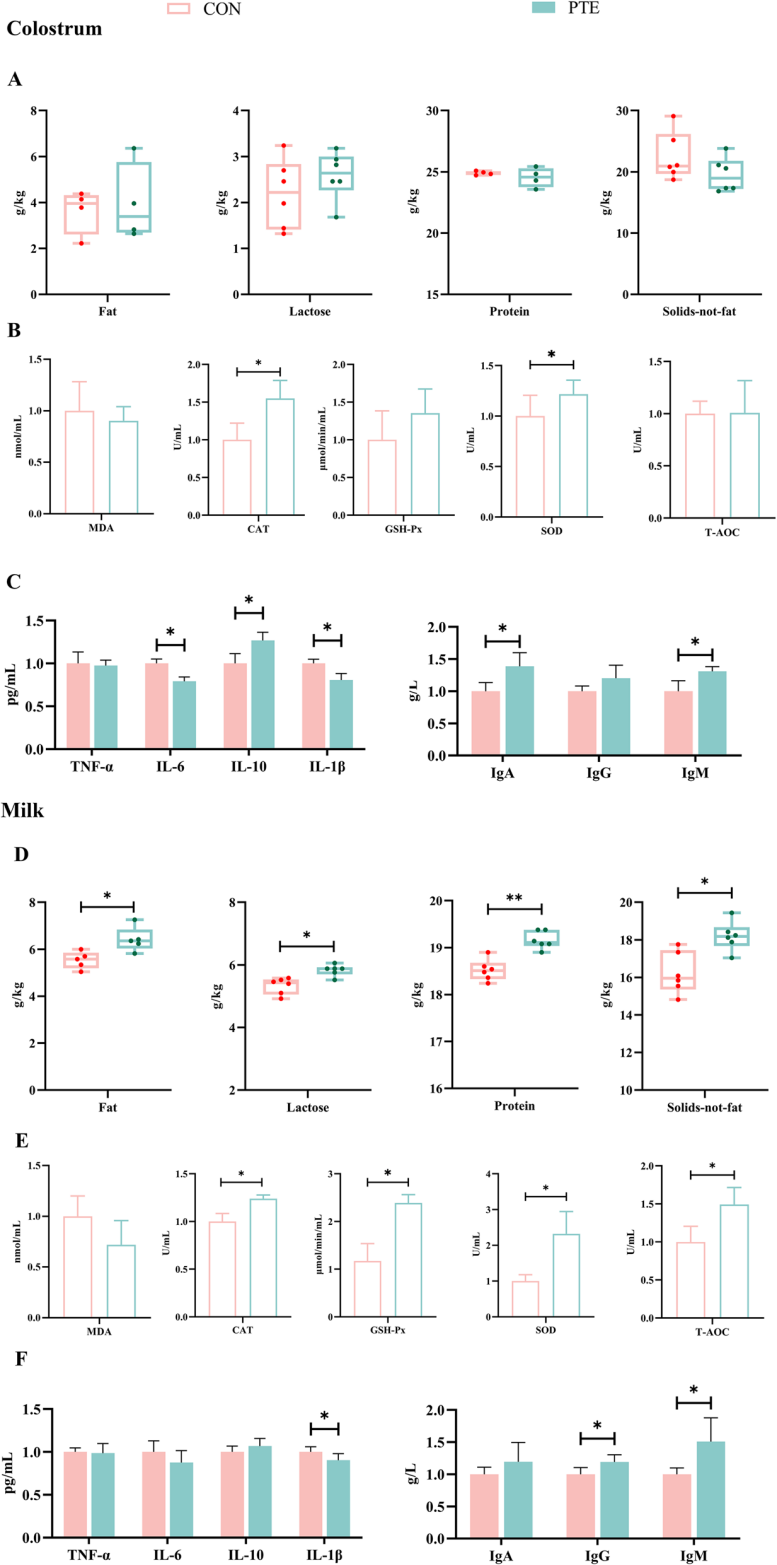
Effect of PTE on milk composition, antioxidant capacity, inflammatory factors and immunoglobulins. **A** Colostrum composition. **B** Colostrum antioxidant capacity. **C** Colostrum inflammatory factor levels and colostrum immunoglobulin levels. **D** Milk composition. **E** Milk antioxidant capacity. **F** Milk inflammatory factor levels and milk immunoglobulin levels. CON: Control group; PTE: Pterostilbene group. Data are expressed as mean ± SD (*n* = 6 for each group). *P<0.05, compared to the control group

The immunocompetence and energy of lactating pups are derived from their breast milk, so we examined changes in the relevant components in colostrum and regular milk. The results are as shown in Fig. 7A. The changes in the fat, lactose, protein and nonfat solids contents of the colostrum were not significant (*P* > 0.05). For milk (Fig. 7D), these nutrients were significantly greater (*P* < 0.05) in the PTE group. Both stages of lactation could be observed because the addition of PTE to the diet increased the fat and lactose contents, which provided a strong energy supply to the lactating pups. In addition, we observed that in colostrum, PTE significantly increased the levels of CAT, SOD, IL-10, IgA, and IgM (*P* < 0.05) and decreased the levels of IL-6, and IL-1β (*P* < 0.05) (Fig. 7B and C). In contrast, in normal milk (Fig. 7E and F), PTE addition significantly increased the CAT, GSH-Px, SOD, T-AOC, IgG and IgM levels (*P* < 0.05) and significantly decreased the IL-1β level (*P* < 0.05). Overall, this study revealed that feeding PTE to lactating mothers increased the nutrient content, antioxidant capacity, and immunoglobulin content of milk and decreased the level of inflammatory factors.

### Effect of PTE on the SCFAs content of sow faeces

**Fig. 8:**
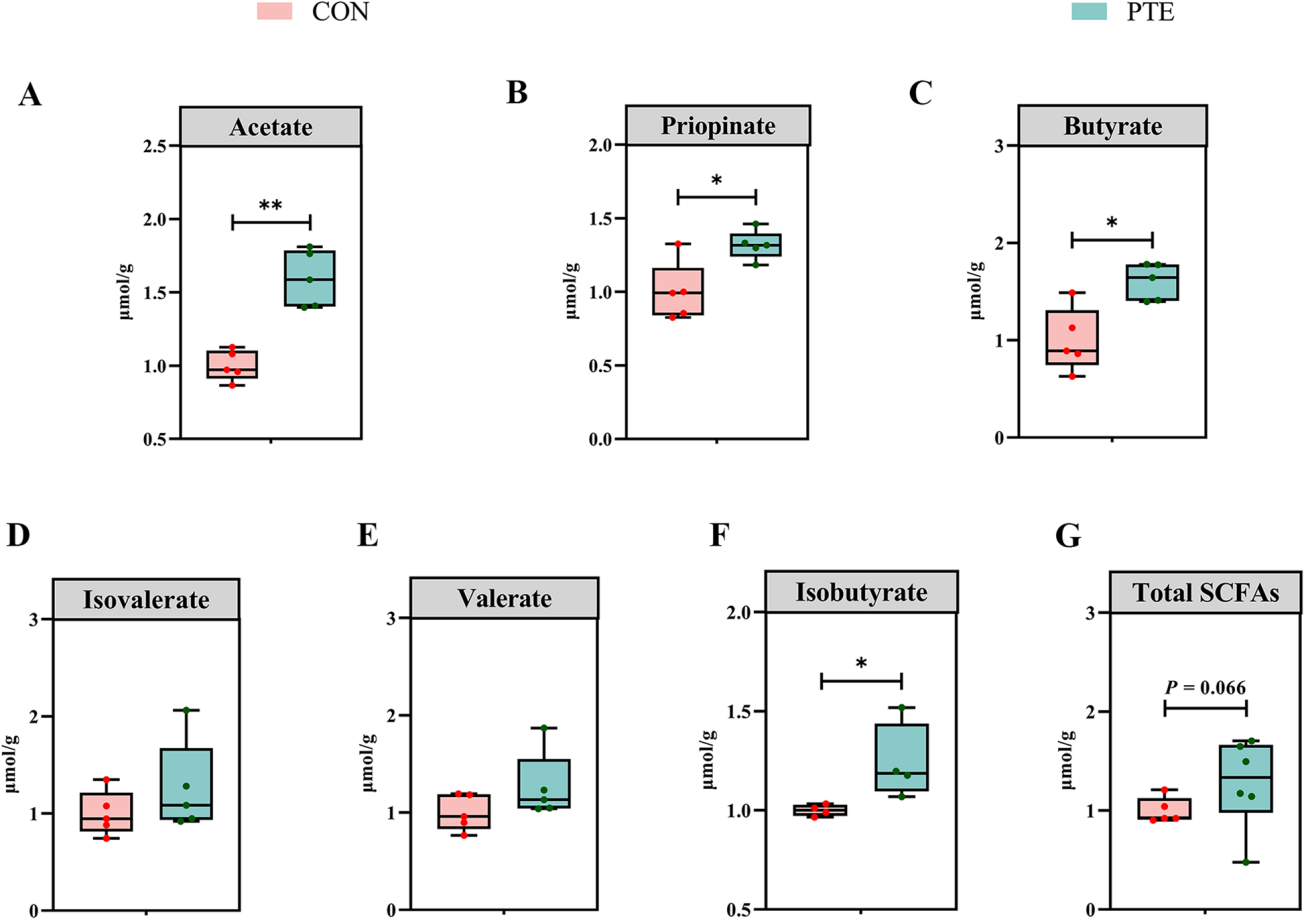
Effect of PTE on fecal SCFAs in sows (**A**–**G**). CON: control group; PTE: Pterostilbene group. Data are expressed as mean ± SD (*n* = 6 for each group). ^*^P < 0.05, compared to the control group

The results are shown in Fig. 8. Compared with those in the CON group, the levels of acetic acid, propionic acid, butyric acid, and isobutyric acid in the PTE group were significantly greater (*P* < 0.05). Unfortunately, there was no significant change in the levels of valeric acid or isovaleric acid (*P* > 0.05), but there was a trend towards a significant increase in the level of total SCFAs (*P* = 0.066).

### Effect of PTE on the faecal microbiota of sows

**Fig. 9:**
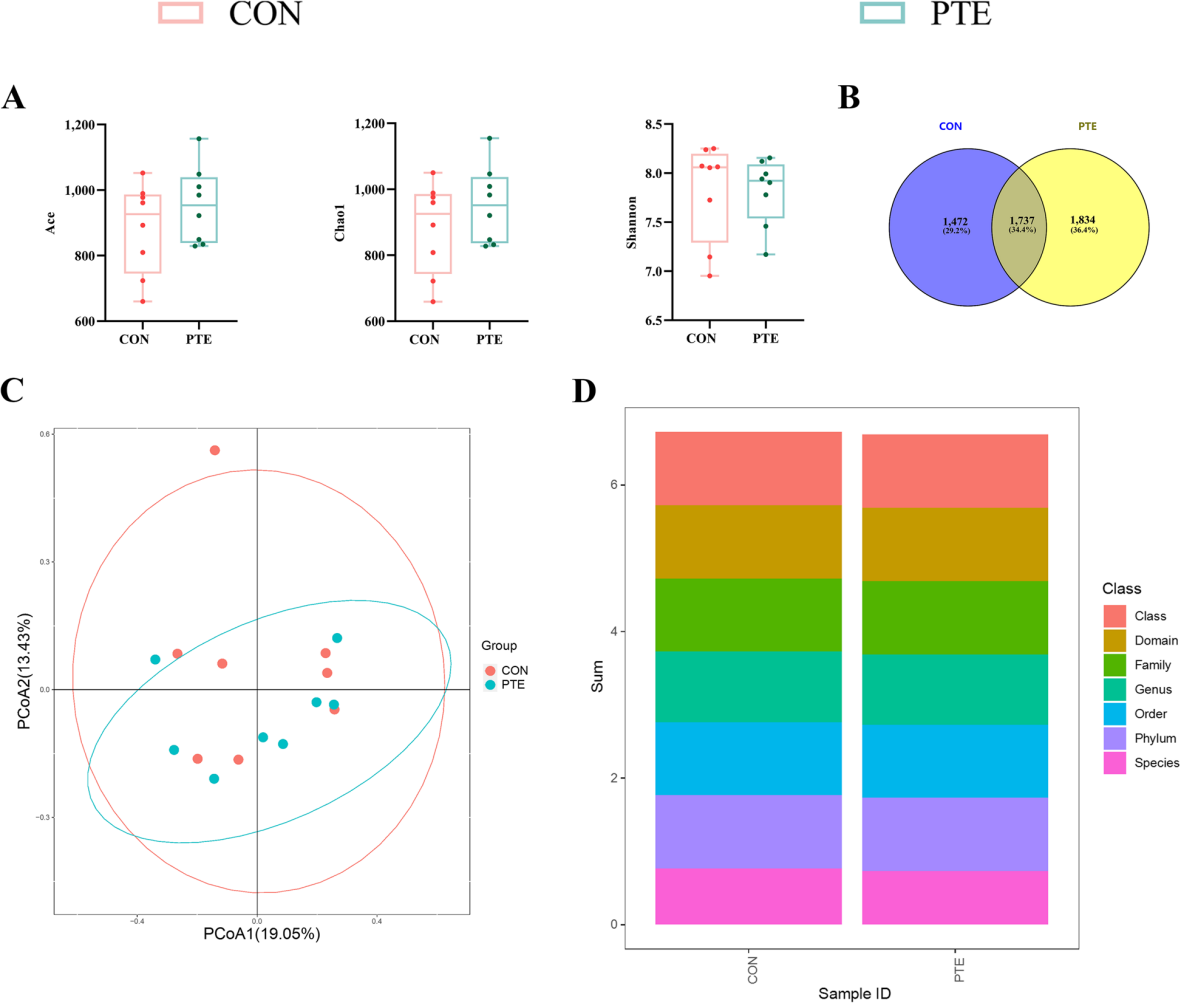
Effect of PTE on the fecal microbiota composition of sows. **A** Fecal microbiota alpha diversity (Ace, Chao, and Shannon). **B** Venn diagram of shared and specific ASVs in the fecal microbiota. **C** Fecal microbiota beta diversity. Data are expressed as mean ± SD (*n* = 8 for each group). ^*^P < 0.05, ^**^P < 0.01 compared to the control group

**Fig. 10:**
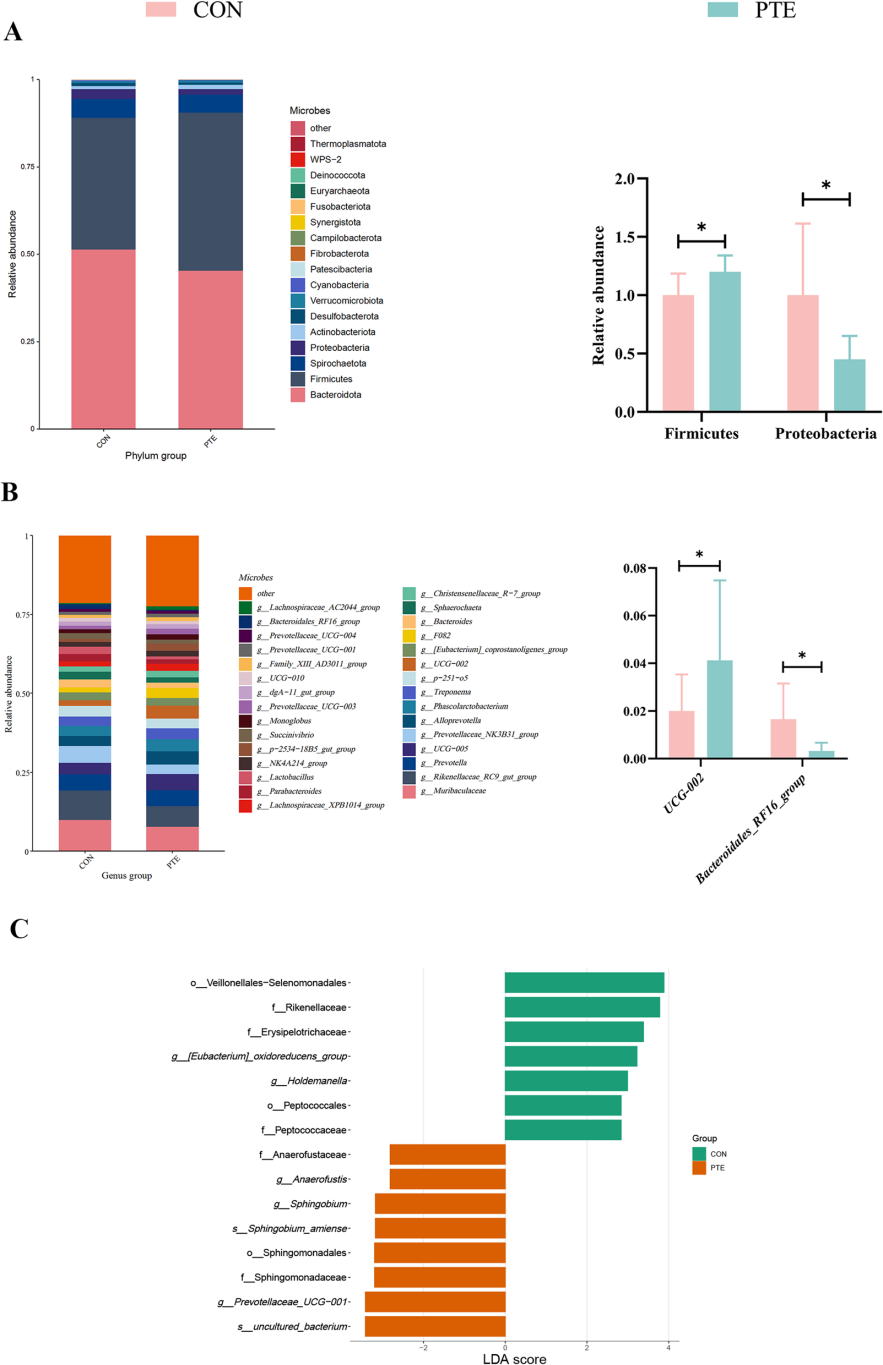
Effect of PTE on the fecal microbiota composition of sows. **A** Relative abundance at the microbiota phylum level. **B** Relative abundance at the genus level of the microbiota. **C** Expression of differences between phyla and genera. CON: control group; PTE: Pterostilbene group. Data are expressed as mean ± SD (*n* = 8 for each group). ^*^P < 0.05, ^**^P < 0.01 compared to the control group

**Fig. 11:**
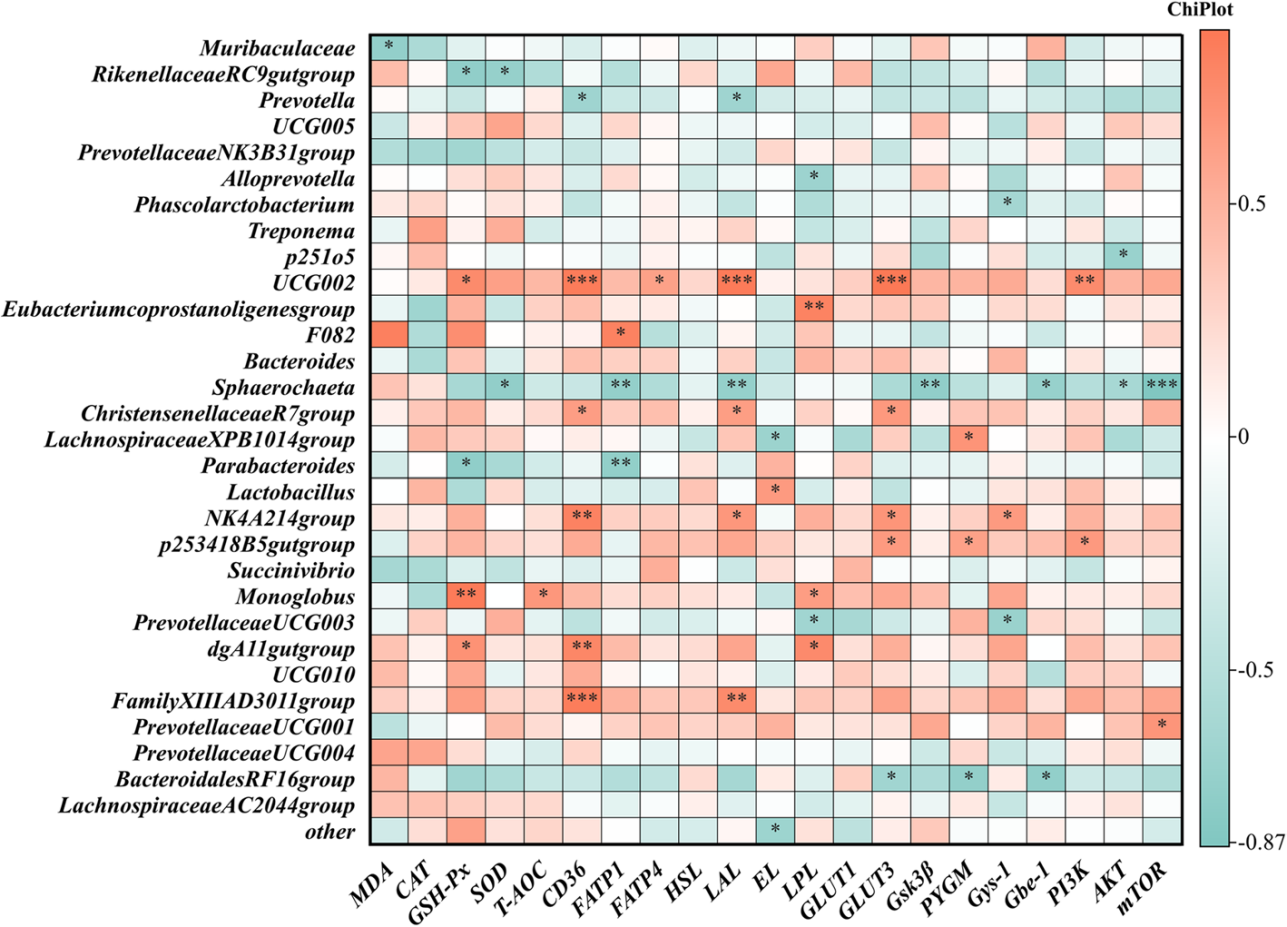
Spearman correlation between nutrient metabolism-related mRNA expression and relative abundance of microbial genera. The R-values are shown in different colors, with red and cyan indicating positive and negative correlation, respectively. ^*^P < 0.05, ^**^P < 0.01 compared to the control group

To investigate the potential mechanism of PTE in mitigating the effects of oxidative stress on energy metabolism and material transport, we analysed the effects of PTE on the microbial constituents of faeces from sows via 16S rRNA analysis. As shown in Fig. 9A, the alpha diversity of the faecal flora in sows (Ace, Chao1, and Shannon) did not differ. A Venn diagram of the shared and specific ASVs in the 2 groups is shown in Fig. 9B. Comparison of the beta diversity (PCoA) of the 2 groups of colonies is shown in Fig. 9C. The 2 groups of faecal flora characterization sequences were annotated for species and biologically classified (Fig. 9D). Differences in the relative abundance of flora between the 2 groups were then explored at the bacterial phylum and genus levels (Fig. 10A–C). The PTE treatment significantly decreased the relative abundance of Proteobacteria and significantly increased the relative abundance of Firmicutes. We also observed significant changes in *UCG-002*, and *Bacteroidales_RF16_group* after the addition of PTE. We further explored the relationships between flora and nutrient metabolism via Spearman correlation analysis. As shown in Fig. 11, *UCG-002* was positively correlated with GSH-Px, *CD36*, *FATP4*, *LAL*, *GLUT3*, and *PI3K*. In contrast, *Bacteroidales_RF16_group* was negatively correlated with *GLUT3*, *PYGM*, and *Gbe-1* (Fig. 11).

## Discussion

During pregnancy, organisms undergo a variety of physiological changes, and accelerated metabolism, high oxygen consumption, and increased release of free fatty acids can generate large numbers of ROS, resulting in an imbalance between oxidation and reduction in mothers [31]. Placental oxidative stress may be associated with pregnancy complications affecting foetal development, which are related to the disruption of placental function and the lack of nutrients and oxygen to the foetus, resulting in hypoplasia [32]. Although several papers on processive oxidative stress in pregnant women have been published in the literature, few have focused on placental function [21]. Therefore, the implementation of nutritional strategies helps maintain redox balance during pregnancy. Safeguarding the function of the placenta has a positive effect on the health of the pregnant woman and the normal development of the foetus.

Sows require higher energy levels and oxygen consumption during the reproductive cycle to meet the metabolic burden of foetal growth, placental development, and increased lactation, which can result in increased ROS production [33]. ROS accumulate as pregnancy progresses, culminating in the process of oxidative stress. Oxidative damage is an important index of animal wellness and welfare. Both MDA (a product of lipid peroxidation) and GSH-Px (an intracellular enzyme that metabolizes away H_2_O_2_ and lipid peroxides) are considered the main markers of oxidative stress occurring in the body [34]. CAT, SOD, and T-AOC constitute the enzymatic antioxidant defence system. SOD and CAT in organisms constitute the first line of antioxidant defence. SOD is responsible for the conversion of O_2_^−^ to H_2_O_2_. CAT converts H_2_O_2_ to H_2_O, thereby eliminating ROS [35]. Previous studies have shown that GSH-Px and T-AOC levels in maternal plasma are lower than those in cord blood in late pregnancy, suggesting that the redox balance in maternal blood is disrupted [36]. In this study, PTE increased mothers’ plasma (7 d before parturition) CAT, SOD, and T-AOC levels. However, when excessive accumulation of ROS breaks the first line of defence, GSH-Px is activated. GSH-Px can reduce various hydroperoxides (ROOH and H_2_O_2_) [37]. An analysis of sow plasma (day of farrowing, 21 days of lactation), revealed that PTE increased the levels of GSH-Px, and T-AOC. Moreover, TC, CHOL, and LDL-C were associated with increased lipid peroxidation [38]. We found that the addition of PTE decreased blood TC levels in mothers 7 d prenatally, and interestingly, at weaning, PTE addition increased TC, and CHOL levels. Therefore, we hypothesized that the antioxidant system of sows just entering late gestation eliminates the ROS produced. As the process of pregnancy progresses, the intensity of maternal metabolism increases and the production of various hydroperoxides increases, disrupting the endogenous antioxidant system. The addition of PTE improved the activity of antioxidant enzymes in the sows.

Redox imbalance is often closely associated with the development of inflammation [39]. Cells are subjected to oxidative damage, thereby promoting the release of arachidonic acid [40]. Arachidonic acid is activated by certain enzymes (cyclooxygenase, lipoxygenase) to transform into inflammatory mediators (e.g., prostaglandins) [41]. Inflammatory mediators act as signalling molecules to recruit neutrophils and macrophages to the site of injury via the body’s circulation, where they produce different inflammatory factors. The decrease in TNF-α and IL-6 levels in mothers’ blood at 7 d before parturition and on the day of parturition, as well as the decrease in IL-6 levels and increase in IL-10 levels in mothers’ blood at the time of weaning, suggest that the addition of PTE reduces the inflammatory response in mothers’ blood. Increased release of inflammatory cytokines has been reported to initiate the defence mechanisms of immune cells [42]. A key component of the defence mechanisms that perform their physiological functions is the production of ROS, which is a vicious cycle [43]. We hypothesized that dietary supplementation with PTE may have terminated this vicious cycle process by increasing the antioxidant capacity of the sows’ blood and reducing the generation of inflammatory factors. Kaiser et al. [44] reported similar outcomes in sows where oxidative stress during gestation altered TNF-α and IL-6 levels. The establishment of maternal–foetal blood circulation contributes to the development of prefoetal immunity. In this study, we found that elevated levels of IgA and IgM at 7 d prenatally, on the day of parturition, and at weaning, as well as elevated IgG at weaning, contributed to the establishment of prenatal foetal immunity.

In maternal–foetal circulation, the placenta transmits maternal blood flow from the uterus to the foetus through the umbilicus, where it acts as the foetus primary organ for exchange of nutrients, gases, and cytokines [45]. The placenta bears the brunt of the challenge of maternal blood redox imbalance. Oxidative stress may interfere with placental nutrient transport and energy metabolism processes leading to adverse pregnancy outcomes [46]. The placenta and foetus cannot develop without lipids, proteins and sugars. Foetuses require large numbers of LCPUFAs for energy metabolism, gene ligand formation and signalling [47]. FATP1, FATP4, and CD36 are the main LCPUFAs transport carriers in the placenta [48]. Foetal protein synthesis requires maternal amino acids [49]. Sodium uncoupled neutral amino acid transport carriers (Snats) and Lats are the major transporters in the placenta [50]. The developing foetus undergoes little gluconeogenesis, so the main source of energy is glucose, and GLUT1 and GLUT3 are the main transport carriers in the placenta [51]. To investigate the impact of oxidative stress on placental transport carriers in depth, we assayed the expression of relevant transporters. The expression of FATP1 and GLUT3 was elevated in the PTE group, which may be another explanation for why the supply of PTE relieved the impacts of oxidative stress in sow blood on placental function. Many types of LCPUFAs and saccharides have been reported to counteract the oxidative damage caused by the placenta to the mother’s blood by promoting the production and release of growth factors in a manner that promotes stable placental function and foetal development [52, 53]. Similar results were reported in a recent study in which PTE inhibited fat deposition in rats fed a high fructose diet by increasing the expression of FATP1, and GLUT3 transporter [54].

The work of transporters is often inextricably linked to energy metabolism processes. No adverse effects of PTE addition on foetal weight were observed, so it was hypothesized that an alternative supply of glucose (e.g., placental glycogen) was available to the foetus. Indeed, PTE upregulated the expression of *PYGM*, and *Gbe1*, and importantly, PTE had no statistically significant effect on *Gys1* or *Gsk3β*. This finding was also confirmed in a study of pregnant female rats [55]. Similarly, LCPUFAs play important roles in foetal growth and development [48]. Endothelial lipase (EL) belongs to the total cholesterol lipase family, activate total cholesterol lipase activity and phosphorylate plasma lipids [56]. Hormone-sensitive lipase (HSL) is an intracellular enzyme with the ability to hydrolyse glycerol and its hydrolysis product, cholesterol esters. Lipoprotein lipase (LPL) is the sole actor in plasma lipoprotein hydrolysis. LAL is the single enzyme capable of hydrolysing neutral lipids in lysosomes [57]. In this study, the addition of PTE increased the expression of *LAL* but did not significantly change the expression of *HSL*, *EL*, or *LPL*. The addition of PTE promoted lysosomal hydrolysis of lipids in placental cells to satisfy the fatty acid demand of the foetus, and the lack of significant changes in other indices indicated that oxidative stress did not disrupt maternal energy metabolism. This finding was also confirmed in a recent study of altered lipase expression due to placental triglyceride accumulation in women with gestational diabetes [58]. *mTOR*, a serine/threonine-specific protein kinase, is a target gene of the *PI3K* pathway and operates as a nutrient sensor during pregnancy to regulate cell growth, and protein transcription [59]. In the present study, PTE addition promoted the expression of *PI3K* and *mTOR*, which was confirmed by the results of Western blotting. These results indicated that PTE supplementation activated the *PI3K* phosphorylation pathway. This finding is consistent with the results obtained by Cui et al. [60] who reported that the addition of leucine to the feed of sows in late pregnancy activated the *PI3K* pathway to promote placental nutrient transport. As a result, we believe that PTE facilitates nutrient transport by activating the *PI3K* phosphorylation pathway to address the challenges posed by oxidative stress in maternal blood to the placenta in the maternal–foetal circulation. However, further testing of this hypothesis is needed.

Shortly after birth, the foetus is exposed to a hostile environment full of pathogens, and the newborn’s immune system and stabilization of the redox state rely on the assistance of breast milk transmission [61]. In this study, PTE improved the activities of CAT and SOD in colostrum and normal milk, in addition to the activities of GSH-Px and T-AOC in milk. It also increased colostrum and milk IgM levels and increased colostrum IgA and milk IgG levels. An increase in the level of immune factors is conducive to equilibrium of the foetal intestinal microecology and the growth of the immune system. A newborn’s energy source is breast milk, and the nutritional content of breast milk directly affects the growth and development of young children. Fat and lactose are the main sources of energy in milk [62]. The addition of PTE to the sows’ rations improved the compositional contents of milk in general and increased the fat and lactose content of milk in particular. Lactose is reportedly synthesized in the secretory alveolar epithelial cells of the mammary gland, and glucose, which is a precursor substance of lactose, is taken up passively from the blood by mammary epithelial cells [63]. Combined with the results of the biochemical tests of the mothers’ blood, a decrease in blood glucose levels and a significant increase in the number of weaned piglets and total weaned litter weights were found. We hypothesized that the addition of PTE could promote the uptake of glucose by mammary epithelial cells from the systemic circulation of the mother, thereby increasing the lactose content of milk and positively affecting the growth and development of piglets.

Pterostilbene is very fat soluble, highly bioavailable (66.9%), and is absorbed by intestinal cells into the body’s circulation [19]. We speculate that PTE may affect the composition of the gut microflora of sows. We discovered that the addition of PTE decreased the partitioning of Proteobacteria and increased the abundance of Firmicutes. An increased abundance of Proteobacteria is a marker of microecological dysregulation and metabolic disorders [64]. Healthy and stable intestinal microecosystems are composed mainly of the phylum Thick-walled Bacteria and the phylum Bacteroidetes. Firmicutes have been reported to positively affect lipid metabolism by inhibiting lipoprotein lipase activity [65]. This may be beneficial for stabilizing oxidative stress-induced metabolic disorders in sows during gestation. At the genus level, the relative abundance of *UCG-002* increased, and the relative abundance of Bacteroidales decreased. These findings are similar to our results. A recent study on the effects of probiotics on the gut microbiota of pregnant women revealed similar findings. Probiotics increase the relative *UCG-002* and *UCG-003* levels in the faeces of pregnant women to promote the production of SCFAs thereby mitigating the effects of stress on the organism [66]. *UCG-002* and *UCG-003* may affect host glucose homeostasis and lipid metabolism by promoting the production of SCFAs to enrich the host energy source [67]. We therefore explored the effects of PTE on SCFAs in faeces. PTE significantly increased the levels of acetate, propionate, butyrate, and isobutyrate. Gut microbes have been reported to draw nutrients primarily from carbohydrates that escape proximal digestion, and ferment to produce SCFAs. SCFAs interact with Gpr41 receptors to enrich host energy sources [68]. We suggest that dietary supplementation with PTE influences the production of SCFAs by promoting the colonization of *UCG-002*, and Firmicutes. SCFAs enter the host’s glucose and fatty acid metabolism, activate the PI3K phosphorylation pathway, and promote the expression of related nutrient transporters. Correlation analysis revealed that *UCG-002* was positively correlated with GSH-Px, *CD36*, *LAL*, *GLUT3*, and *PI3K*, which confirms this speculation. In short, maternal dietary supplementation with PTE alters the bacterial biota, enriches the beneficial flora, improves the antioxidant capacity of the host, and increases maternal metabolism to meet the energy needs of the foetus.

## Conclusion

In conclusion, dietary supplementation with 500 mg/kg PTE prevents processivity oxidative stress in sows by increasing the antioxidant capacity of blood and milk during gestation and lactation. In this way, the enzymes involved in fatty acid and glucose metabolism are protected from stress and metabolic dysfunction of the placenta is prevented. The normal growth and development of the foetus were protected. This study provides a theoretical basis for further exploration of PTE as a dietary supplement during pregnancy to safeguard the health of sows during pregnancy and rapid recovery after farrowing.

## Supplementary Information

**Additional file** **1. Fig. S1.** Diagram of the pterostilbene molecular structure used in this study. **Table S1.** Sow gestation and lactation feed formulations used in this study. **Table S2.** The qRT-PCR primer sequences used in this study. **Table S3.** Information about the antibodies used in this study.

## Data Availability

The raw data analyzed in this study are available from the corresponding author upon request, subject to the provision of a reasoned justification.

## Abbreviations

AKT: Protein kinase B
ALB: Albumin
ALP: Alkaline phosphatase
ALT: Alanine aminotransferase
AST: Aspartate aminotransferase
BUN: Blood urea nitrogen
CAT: Catalase
CHOL: Cholesterol
CON: Control group
EL: Endothelial lipase
FATP1: Fattyacidtransportprotein1
FATP3: Fattyacidtransportprotein3
Gbe-1: Glycogen synthase 1
GLOB: Globulin
GLU: Glucose
GLUT1: Glucose transporter 1
GLUT3: Glucose transporter 3
GSH-Px: Glutathione peroxidase
GSK3β: Recombinant glycogen synthase kinase 3 beta
Gys-1: Glycogen synthase 1
HDL-C: High density lipoprotein cholesterol
HSL: Hormone-sensitive lipase
IF: Immunofluorescence
IL-10: Interleukin 10
IL-1β: Interleukin 1β
IL-6: Interleukin 6
LAL: Lysosomal acid lipase
LD: Lactate dehydrogenase
LDL-C: Low density lipoprotein cholesterol
IgA: Immunoglobulin A
IgG: Immunoglobulin G
IgM: Immunoglobulin M
LPL: Lipoprotein lipase
MDA: Malondialdehyde
mTOR: Mammalian target of rapamycin
PI3K: Phosphatidylinositol-3-kinase
PTE: Pterostilbene
PYGM: Glycogen phosphorylase M
SNAT1: Recombinant sodium coupled neutral amino acid transporter 1
SNAT3: Recombinant sodium coupled neutral amino acid transporter 3
SOD: Superoxide dismutase
T-AOC: Total antioxidative capacity
TBA: Total bile acids
TC: Total cholesterol
TG: Triglyceride
TNF-α: Tumor necrosis factor α
TP: Total protein
UA: Uric acid
