# Correction: Dietary supplementation with pterostilbene activates the PI3K-AKT-mTOR signalling pathway to alleviate progressive oxidative stress and promote placental nutrient transport

**DOI:** 10.1186/s40104-024-01124-2

**Published:** 2024-11-25

**Authors:** Mingming Cao, Liyun Bai, Haoyun Wei, Yantong Guo, Guodong Sun, Haoyang Sun, Baoming Shi

**Affiliations:** College of Animal Science and Technology, Northeast Agricultural University, Harbin, 150030 PR China

**Correction** **:** **J Animal Sci Biotechnol 15, 133 (2024)**

**https://doi.org/10.1186/s40104-024-01090-9**

Following publication of the original article [1], the authors reported errors in the legend of Fig. 7 and the *P* value of Fig. 8G (0.66 should be corrected to 0.066).

The originally published legend of Fig. 7 was:

Effect of PTE on milk composition, antioxidant capacity, inflammatory factors and immunoglobulins. **A** Colostrum composition. **B** Colostrum antioxidant capacity. **C** Colostrum inflammatory factor levels. **D** Colostrum immunoglobulin levels. **E** Milk composition. **F** Milk antioxidant capacity. **G** Milk inflammatory factor levels. **H** Milk immunoglobulin levels. CON: control group; PTE: Pterostilbene group. Data are expressed as mean ± SD (*n* = 6 for each group). ^*^P < 0.05, compared to the control group.

The corrected legend of Fig. 7 should read:

Effect of PTE on milk composition, antioxidant capacity, inflammatory factors and immunoglobulins. **A** Colostrum composition. **B** Colostrum antioxidant capacity. **C** Colostrum inflammatory factor levels and colostrum immunoglobulin levels. **D** Milk composition. **E** Milk antioxidant capacity. **F** Milk inflammatory factor levels and milk immunoglobulin levels. CON: Control group; PTE: Pterostilbene group. Data are expressed as mean ± SD (*n* = 6 for each group). ^*^P < 0.05, compared to the control group.

The originally published Fig. 8 was:

**Fig. 8:**
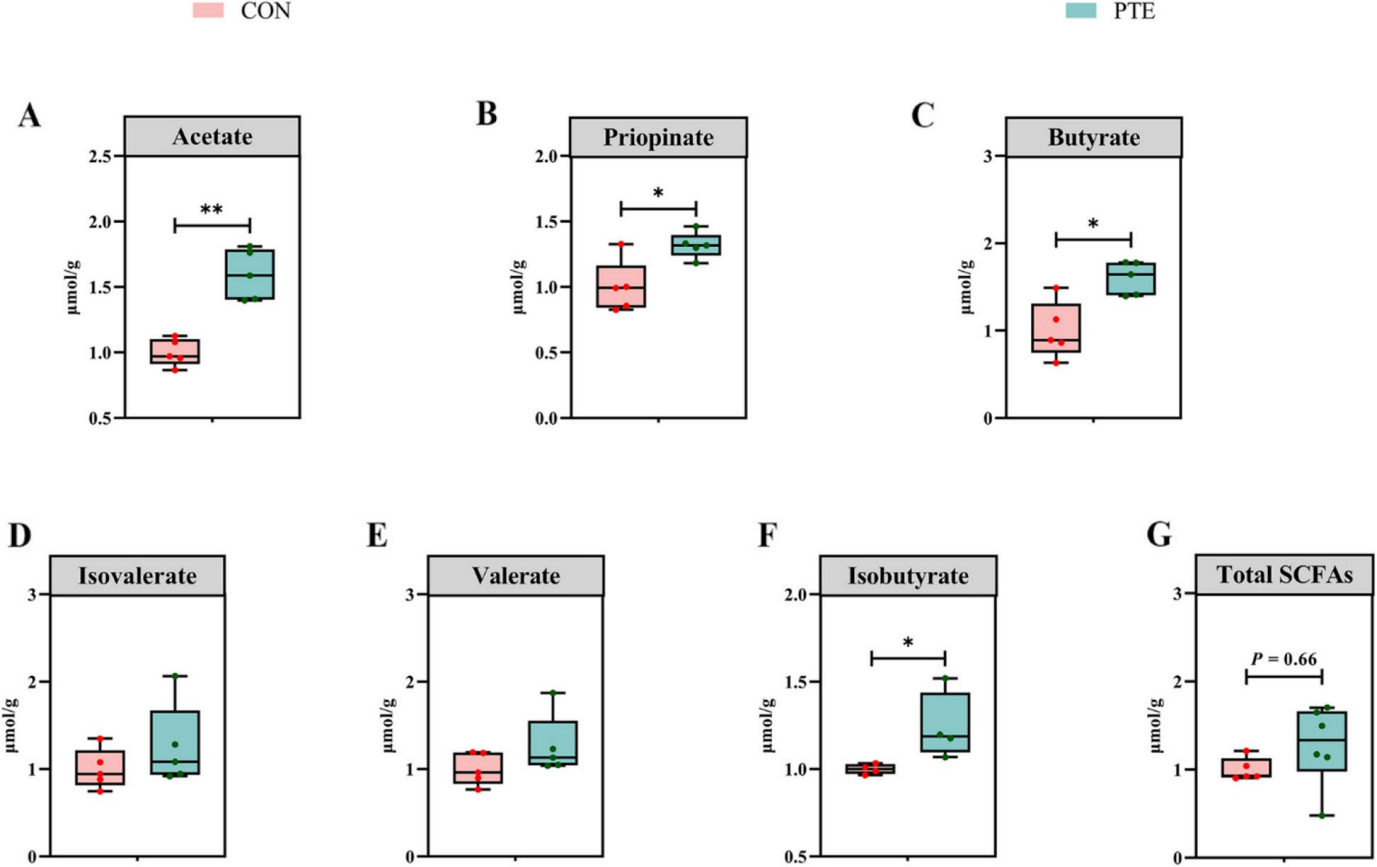
Effect of PTE on fecal SCFAs in sows (**A**–**G**). CON: control group; PTE: Pterostilbene group. Data are expressed as mean ± SD (*n* = 6 for each group). ^*^P < 0.05, compared to the control group

The corrected Fig. 8 should read:

**Fig. 8:**
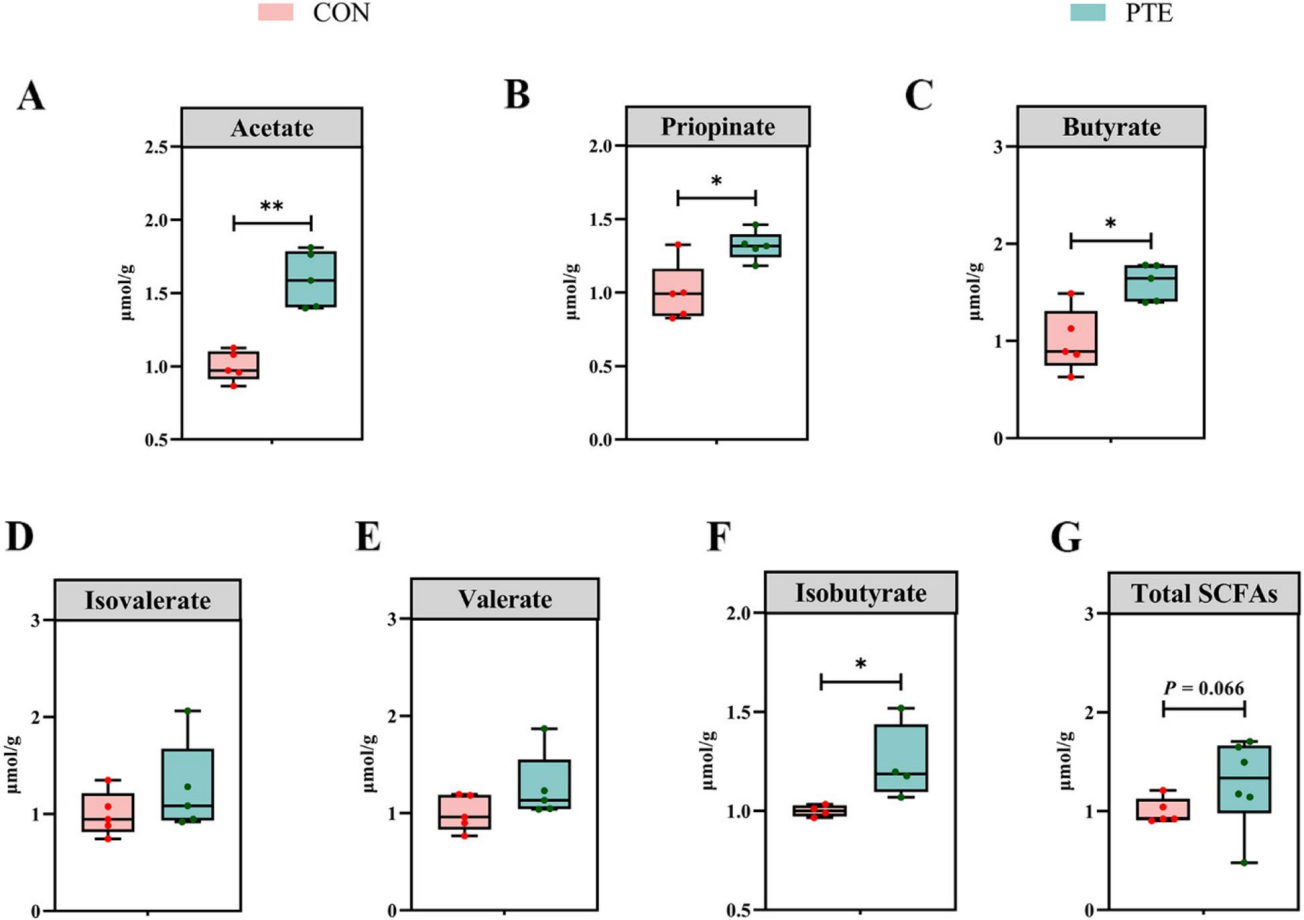
Effect of PTE on fecal SCFAs in sows (**A**–**G**). CON: control group; PTE: Pterostilbene group. Data are expressed as mean ± SD (*n* = 6 for each group). ^*^P < 0.05, compared to the control group

The original article [1] has been updated.
