# Supplementary material for: Dietary supplementation with pterostilbene activates the PI3K-AKT-mTOR signalling pathway to alleviate progressive oxidative stress and promote placental nutrient transport
Source: J Anim Sci Biotechnol. 2024 Oct 6;15:133. doi: 10.1186/s40104-024-01090-9 (PMC11456245; doi:10.1186/s40104-024-01090-9)
Supplement: Supplementary file 1 — Additional file 1. Fig. S1. Diagram of the pterostilbene molecular structure used in this study. Table S1. Sow gestation and lactation feed formulations used in this study. Table S2. The qRT-PCR primer sequences used in this study. Table S3. Information about the antibodies used in this study. [file 40104_2024_1090_MOESM1_ESM.docx]

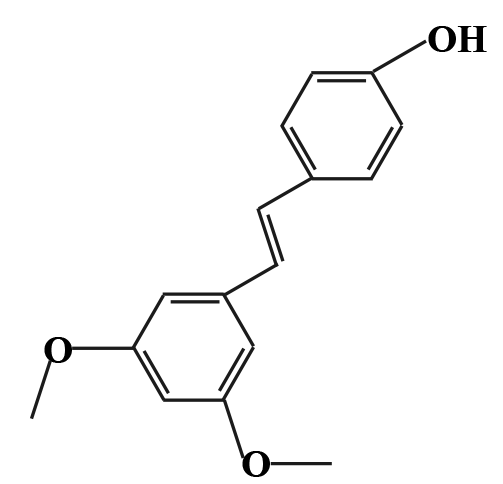


**Fig. S1** The chemical structure of pterostilbene (C_16_H_16_O_3_)

**Table S1** Basic diet ingredients and nutrient levels of basal diets (as-fed basis)

| **Items, %** | **Gestation** | **Lactation** | |
| --- | --- | --- | --- |
| Basic diet ingredients |  |  | |
| Corn | 66.00 | 60.00 | |
| Corn DDGS | 1.50 | 4.60 | |
| Soybean meal | 22.00 | 24.00 | |
| Rice bran meal | 5.10 | 4.30 | |
| Fish meal | 1.00 | 2.00 | |
| Soybean oil | 1.00 | 2.00 | |
| Calcium hydro phosphate | 1.00 | 1.00 | |
| Mountain flour | 0.85 | 0.65 | |
| NaCl | 0.55 | 0.45 | |
| Premix^a^ | 1.00 | 1.00 | |
| Total | 100 | 100 | |
| Nutritional level^b^ | | |  |
| Digestible energy, Mcal/kg | 3.38 | 3.43 | |
| Crude protein | 17.89 | 19.67 | |
| Lysine | 0.88 | 1.06 | |
| Methionine | 0.27 | 0.30 | |
| Threonine | 0.57 | 0.62 | |
| Tryptophan | 0.21 | 0.22 | |
| Calcium | 0.74 | 0.80 | |
| Total phosphorus | 0.69 | 0.72 | |
| Available phosphorus | 0.36 | 0.40 | |

^a^ Provided the following per kilogram of diet: 8,000 IU Vitamin A; 2,000 IU Vitamin D_3_; 140 mg Vitamin E; 2 mg Vitamin K_3_; 2.4 mg Vitamin B_1_; 8.5 mg Vitamin B_2_; 4.5 mg Vitamin B_6_; 0.03 mg Vitamin B_12_; 20 mg pantothenic acid; 3.5 mg folic acid; 0.52 mg biotin; 28 mg niacin; 100 mg Zn; 20 mg Cu; 80 mg Fe; 25 mg Mn; 0.4 mg I; 0.3 mg Se

^b^ Nutritional levels were calculated value

**Table S2** Primers and PCR products for gene expression analysis by Real-Time qPCR

| **Gene** | **Primer sequence (5´→3´)** | **Fragments sizes, bp** | **Accession No.** |
| --- | --- | --- | --- |
| *β-actin* | F: GGCACCACACCTTCTACAACGAG | 102 | XM_003124280.5 |
|  | R: TCATCTTCTCACGGTTGGCTTTGG |  |  |
| *FATP1* | F: GTGCTGAGTCGCCTGCTTGG | 82 | XM_021076151.1 |
|  | R: CCATGCCTGCTTTGCCCTCTAC |  |  |
| *FATP4* | F: CTACCACACGGCAGGCAACATC | 108 | XM_013993903.2 |
|  | R: ACACAATCATCCCAGAACCGAGAAG |  |  |
| *CD36* | F: TACAGCCCAATGGTGCCATCTTTG | 86 | XM_021102279.1 |
|  | R: TGCCACAGCCAGATTGAGAACAG |  |  |
| *SNAT1* | F: AAGCAGAAGAAGTCTCACGAACAGC | 141 | XM_003355629.4 |
|  | R: GCAAAGGCGAGTCCCAGAATCC |  |  |
| *SNAT2* | F: AGCACCTTTACACAGCCCACAAC | 127 | XM_013997964.2 |
|  | R: AGGTCAGAATTGGCACAGCATAGAC |  |  |
| *LAT2* | F: CACCCTCAGTCAAACCCTCT | 168 | XM_003128550.6 |
|  | R: GGAACTCCTCCAGCTGACTT |  |  |
| *GLUT1* | F: ACGGTGCTCCTGGTCCTGTTC | 129 | XM_021096908.1 |
|  | R: CTCGGGTGTCTTGTCGCTTTGG |  |  |
| *GLUT3* | F: CCTGGGCCGATTGGTTATTG | 139 | XM_021092392.1 |
|  | R: CAGAATCCCGATGACGATGC |  |  |
| *HSL* | F: TTGAAATGCCACTGACTGCTGAC | 132 | NM_214315.3 |
|  | R: GCTCCTCACTGTCCTGTCCTTC |  |  |
| *LAL* | F: CATGGCTTGCTGGCAGATTCC | 132 | XM_021071782.1 |
|  | R: GTGTTTCCGAGACCAGGTGTTTC |  |  |
| *EL* | F: AAGAGAGAAAGGAGCCAATGTTGTG | 85 | XM_021094488.1 |
|  | R: CCTCGTGTTATTGACCGCATCTAC |  |  |
| *LPL* | F: AAGATCAGAGTGAAGGCAGGAGAG | 125 | XM_021072174.1 |
|  | R: TTCAGAGACTTGTCGTGGCATTTC |  |  |
| *GSK3B* | F: TGTGTTGGCTGAACTGTTGCTAG | 112 | XM_021068187.1 |
|  | R: TCTAATTTGCTCCCTTGTTGGTGTC |  |  |
| *PYGM* | F: ATCCGCCGCTTCAAGTCCTC | 149 | XM_003122588.5 |
|  | R: TCCTCATCCACCAGAATCCTCATC |  |  |
| *Gbe-1* | F: TGTGACTCGTGAAGGTGATAATGTG | 96 | XM_021070783.1 |
|  | R: TTTGGCTTCTTTGGTTTGGAATGC |  |  |
| *Gys-1* | F: CCAGTATCTCCACCAACCTCTCC | 89 | NM_001195508.1 |
|  | R: GTCCAGAATGTAGATGCCGTAAGC |  |  |
| *PI3K* | F: GGCAATGTGGAGCAGATGAAGG | 109 | XM_021102206.1 |
|  | R: GGTAGAGCAGGAGGAAGTGGTC |  |  |
| *AKT* | F: GGCAATGTGGAGCAGATGAAGG | 124 | XM_021081501.1 |
|  | R: GGTAGAGCAGGAGGAAGTGGTC |  |  |
| *mTOR* | F: CGCCTATTTGCCTATCCTGACACTC | 83 | NM_214301.2 |
|  | R: GCACGGAAGGGACAGTTCACAG |  |  |

**Table S3 Information for primary and secondary antibodies**

| **Primary antibodies** | **Dilution ratio** | **Source of antibodies** | **Cat. No.** | **Clone type** |
| --- | --- | --- | --- | --- |
| Rabbit Anti-PI3K P110β antibody | 1:1,000 | Wanleibio, Shenyang, China | WL03380 | Polyclonal antibody |
| Rabbit Anti-Phospho-Akt antibody | 1:500 | ABclonal, Wuhan, China | AP1208 | Polyclonal antibody |
| Rabbit Anti-Pan-Akt antibody | 1:500 | ABclonal, Wuhan, China | A18675 | Polyclonal antibody |
| Rabbit Anti-FATP1 antibody | 1:200 | ABclonal, Wuhan, China | A12847 | Polyclonal antibody |
| Rabbit Anti-GLUT3 antibody | 1:200 | ABclonal, Wuhan, China | A8150 | Polyclonal antibody |
| GAPDH Rabbit mAb | 1:10,000 | Servicebio, Wuhan, China | GB15004 | Polyclonal antibody |
| DAPI |  | Servicebio, Wuhan, China | G1012 |  |
|  |  |  |  |  |
| **Secondary antibody** | **Dilution ratio** | **Source of antibodies** | **Cat. No.** |  |
| HRP Goat Anti-Rabbit IgG (H+L) | 1:3,000 | Servicebio, Wuhan, China | GB23303 |  |
